# Exploration of Japanese women seeking acupuncture for menopausal symptoms: a preliminary study

**DOI:** 10.15406/ijcam.2023.16.00674

**Published:** 2023-12-18

**Authors:** Junko Hirota, Miho Takayama, Morihiro Nasu, Judith M. Schlaeger, Hiroyoshi Yajima, Nobuari Takakura

**Affiliations:** 1 Department of Acupuncture and Moxibustion, Tokyo Ariake University of Medical and Health Sciences, Japan; 2 Haplus Acupuncture Clinic, Japan; 3 Department of Human Development Nursing Science, College of Nursing, University of Illinois Chicago, USA

**Keywords:** acupuncture, menopause, musculoskeletal symptoms, simple menopause index, gynecology

## Abstract

Menopausal symptoms may affect every aspect of women’s lives. There are no studies that examine the rate of menopausal women who seek acupuncture for their complaints, particularly muscle stiffness and aches, headaches, fatigue, and depression, which are indications for acupuncture, in Japan. The aim of this preliminary study was to explore the rate of Japanese women in menopause who sought acupuncture for the treatment of their general complaints, and to what extent acupuncture reduced their menopausal symptoms. 29 Japanese women, ages 40 to 59, received three individualized acupuncture treatments at 7 acupuncture clinics in Tokyo and surrounding suburbs. Menopausal symptoms were assessed by the Simple Menopause Index (SMI) which consisted of 10 symptoms from three categories: vasomotor, psychoneurological and musculoskeletal symptoms to determine if women were in menopause. Fifteen of 29 Japanese women had an SMI score greater than or equal to 26, suggesting that they were in menopause. Menopausal symptoms were reduced with individualized acupuncture treatments, exclusively due to improvement of musculoskeletal symptoms. Vasomotor and psychoneurological symptoms were not improved. These results suggest Japanese women in menopause seeking acupuncture may benefit from musculoskeletal symptom relief such as fatigue, chronic neck pain, and low back pain. Considering these results, acupuncturists may advise them to be evaluated by and inform gynecologists of their intention to use acupuncture to treat menopausal symptoms. Future studies focused on improvement of musculoskeletal symptoms and possibly vasomotor and psychoneurological symptoms with larger sample sizes are necessary.

## Introduction

In Japan, acupuncturists often treat women around the age of 50 who experience menopausal symptoms such as hot flashes, sleep disturbance, fatigue, joint/muscle pain, and anxiety.^1,2^ Women worldwide experience some or all of these symptoms around the age of 50 marked by changes in hormonal status and cessation of the menstrual cycle.^2^ Quality of life, health status, and work productivity can be greatly affected by these common menopausal complaints.^3,4^

In Japan, many women being treated with acupuncture do not realize that they are experiencing menopausal symptoms. Therefore, it is important for Japanese women in menopause and their gynecologists to know the rate of women suffering from menopausal symptoms who seek acupuncture for relief, and to what extent their symptoms reduce with acupuncture. There are no Japanese studies that have reported this information. The purpose of this preliminary study was to explore the rate of menopause in Japanese women who used acupuncture and to determine if acupuncture reduced these symptoms.

## Material and methods

All study objectives and methods were explained, and informed consent was obtained from all participants. This study was approved by the Ethics Committee of Tokyo Ariake University of Medical and Health Sciences (approval no. 282).

### Participants and Acupuncture treatments

Participants were 29 Japanese women ages 40 to 59 (mean age: 47.6±3.6 years old) who received acupuncture from May to August 2019. Seven acupuncture clinics with eight acupuncturists participated in this study. Participants received three acupuncture treatments each. Menopausal symptoms were assessed before the first and fourth treatment. All treatments were individualized by each acupuncturist according to their assessment of the participant on each treatment day.

### Simple Menopause Index (SMI)

Menopausal symptoms were assessed using the Simple Menopause Index (SMI) which was developed in Japan.^5,6^ The SMI is a questionnaire consisting of 10 symptoms rated on 4 intensity levels (absent, mild, moderate, or severe), which are considered to reflect estrogen levels in menopausal women.^5,7–9^ The total score for the SMI is one hundred points with a separate subscale score for each of 3 symptom categories. The 10 symptoms are vasomotor (4 symptoms, 46 points), psychoneurological (4 symptoms, 40 points) and musculoskeletal (2 symptoms,14 points) symptoms.^5,8^ Women with a total score of 26 or higher on the SMI are considered to have menopausal symptoms and in Japan it is recommended they have a gynecological exam.^10^ The SMI contains simple and easy questions,^5,7^ and its reproducibility has been established.^6–9^ The SMI is widely used in Japan.^5–7^

### Data analysis

The ten symptoms were summed to achieve a total subscale score (vasomotor, psychoneurological and musculoskeletal symptoms) for each woman.^5–9^ The SMI subscale scores before the first treatment and at the fourth treatment were compared using Wilcoxon’s signed rank test.

## Results

**Figure 1:**
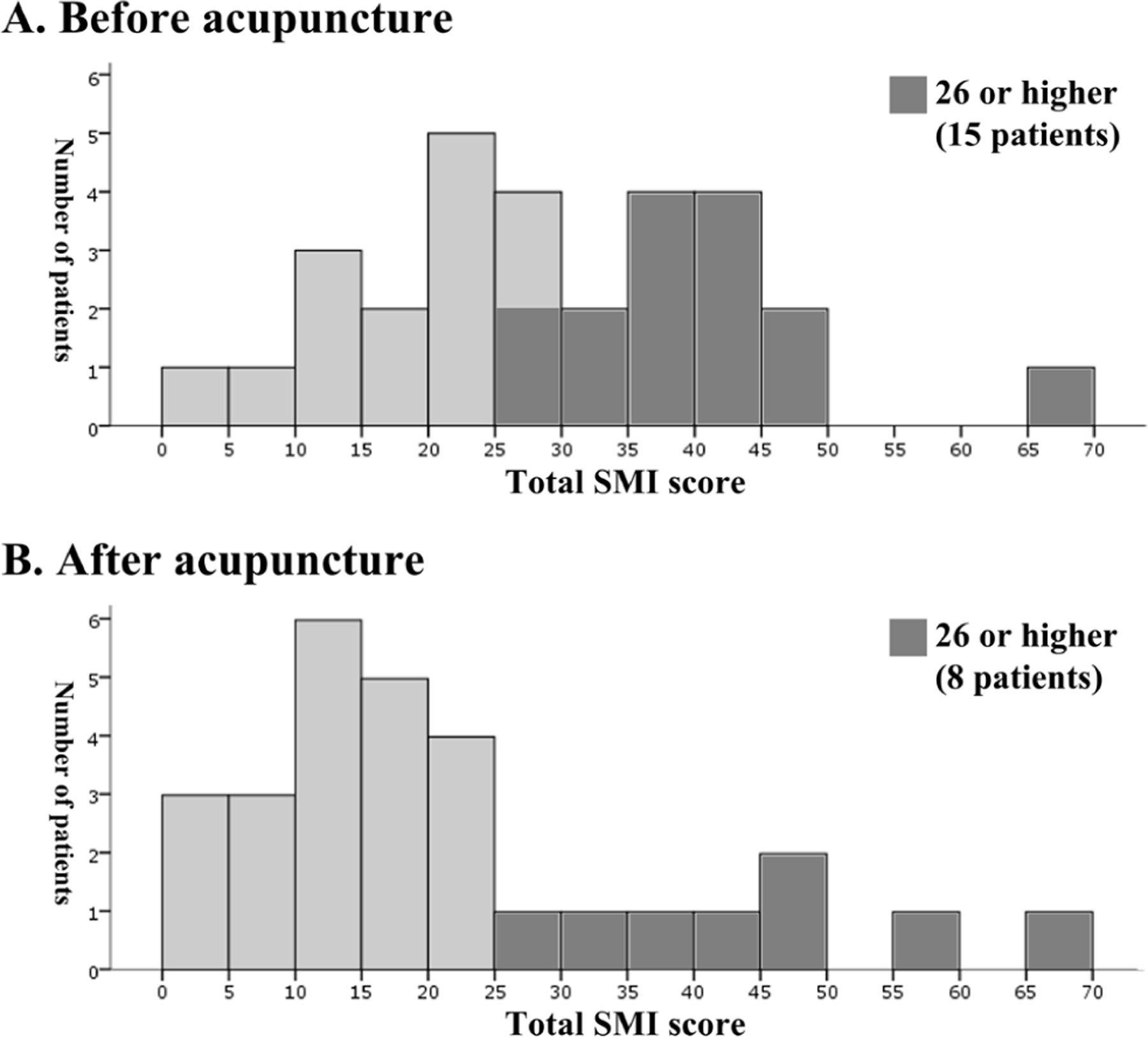
Histograms of total Simple Menopause Index (SMI) subscale score (A) before the first acupuncture treatment (visit 1) and (B) after three acupuncture treatments (visit 4).

**Figure 2:**
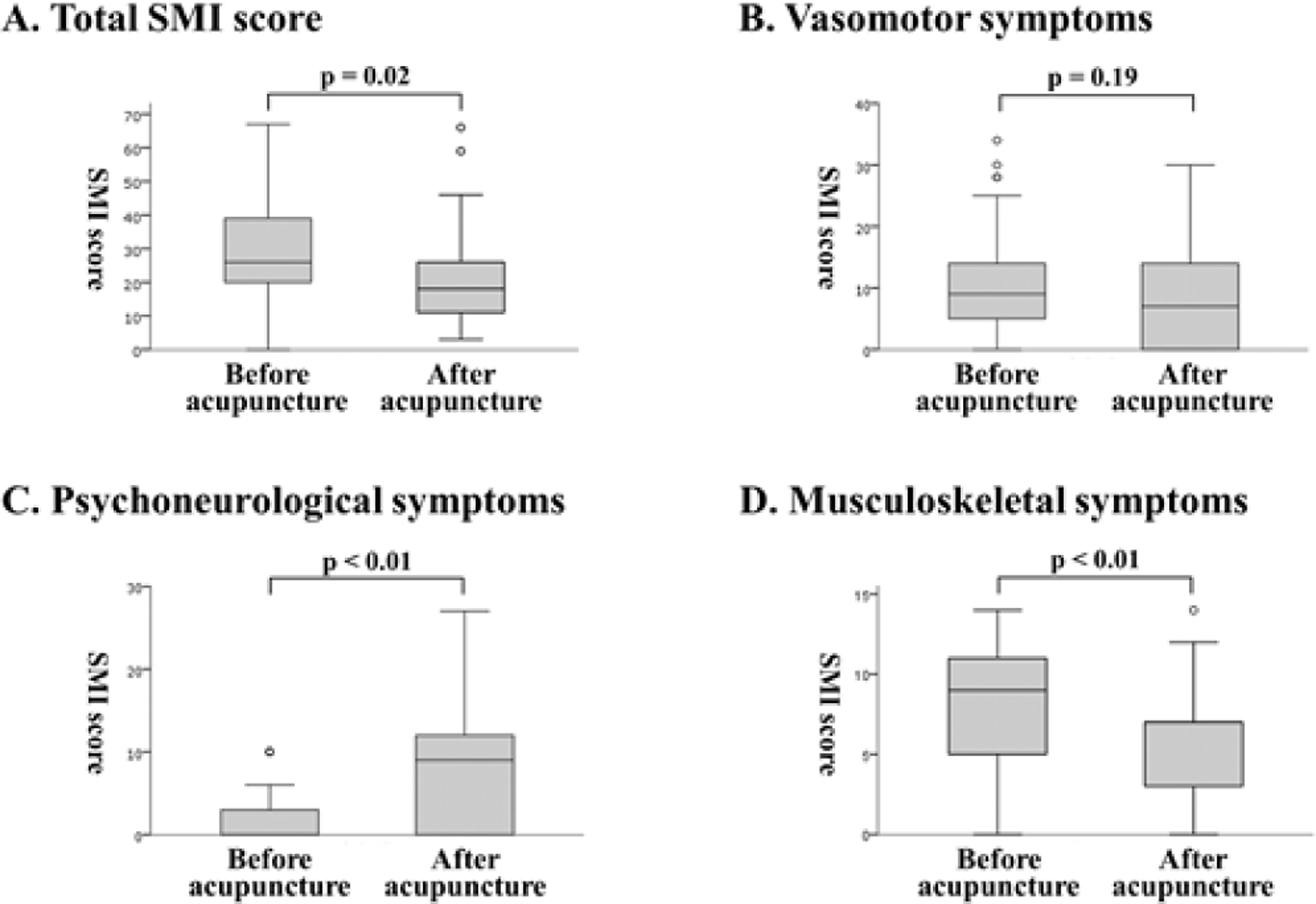
Changes in (A) total Simple Menopause Index (SMI) score, and SMI scores for (B) vasomotor, (C) psychoneurological, and (D) musculoskeletal symptoms after acupuncture treatments.

The mean time from the first visit to the fourth visit for 29 women was 6.9±3.0 weeks. Fifteen of 29 (52%) women had SMI subscale scores greater than or equal to 26. These participants were considered to have menopausal symptoms before the first acupuncture treatment. At the fourth visit before treatment, 7 (46.7%) of the 15 participants SMI scores reduced to below 26 (Figure 1), which is considered normal. For all 29 women, the mean (median)±SD SMI score of 28.8 (26)±14.2 before the first acupuncture treatment reduced significantly to 22.7 (18)±16.4 before the fourth acupuncture treatment (p=0.021). There was also a significant reduction in the SMI subscale score for the musculoskeletal symptoms category (p< 0.01). A significant increase in SMI scores was present in psychoneurological symptoms (p<0.01) and no significant change in the vasomotor symptoms was observed after the three acupuncture treatments (p=0.19) (Figure 2).

## Discussion

Approximately half of the Japanese women, n=15 (52%) who received acupuncture in our preliminary study may have been in menopause according to the SMI. It may be necessary for Japanese acupuncturists to differentiate between menopausal symptoms and Japanese women who simply have general complaints unassociated with menopause by using the SMI. The SMI reflects menopausal symptoms and can be easily checked in a clinical setting.^5,7^

In our small sample size, there was a reduction and therefore an improvement in menopausal symptoms using the SMI subscale scores, but it was exclusively attributed to musculoskeletal symptoms. Prior acupuncture studies on menopause in general focused mostly on vasomotor symptoms and insomnia.^11–15^ The current results did not support previous studies that showed acupuncture reduces hot flashes, insomnia, and depression. ^11–16^ This may be due to the small sample size. Also, our sample showed an increase in psychoneurological symptoms. We speculated unfavorable events that women reported to the acupuncturists may have had a significant impact on women’s mental states during the treatment period. For musculoskeletal symptoms in menopause, the prevalence of aches or stiff joints in menopausal women ranged from 41% to 57%, and stiffness and soreness were greater than 41%.^17–19^ The reduction in musculoskeletal symptoms might be due to the acupuncture treatments, although the placebo effect cannot be completely ruled out. Results of this study suggest that more research may need to focus on the reduction of musculoskeletal symptoms such as fatigue, chronic neck pain, low back pain, and other arthralgias in Japanese menopausal women. Because musculoskeletal symptoms among Japanese women in menopause may be related to changes in hormonal status, Japanese acupuncturists should refer these women to a gynecologist to ensure they are candidates for acupuncture.

Limitations of this preliminary study include non-randomization, small sample size, no placebo control group, and a lack of a standardized acupuncture treatment protocol. Current results show that there is a need to conduct a double-blind placebo controlled, powered, randomized controlled trial to determine the efficacy of acupuncture using the SIM to measure symptoms of menopause in Japanese women.

The number of menopausal women is expected to increase rapidly worldwide from 467 million in 1990 to 1.2 billion in 2030.^20^ This suggests acupuncture treatments for menopausal women may greatly increase and become an important integrative therapy. Acupuncture, with its low side effects profile, has potential for treating musculoskeletal and potentially other related symptoms of women in menopause.^11,12,15^

## Conclusion

Menopausal symptoms in Japanese women, who visited acupuncturists to treat multiple ‘general complaints’ and were assessed as menopause by Simple Menopause Index, were improved exclusively due to reducing musculoskeletal symptoms but neither vasomotor nor neuropsychiatric symptoms. Future rigorous studies should be conducted to verify the genuine efficacy or effectiveness of acupuncture on menopausal symptoms.

## Abbreviations

SMI: simple menopause index
SD: standard deviation
